# Reinforcement learning for patient-specific optimal stenting of intracranial aneurysms

**DOI:** 10.1038/s41598-023-34007-z

**Published:** 2023-05-02

**Authors:** E. Hachem, P. Meliga, A. Goetz, P. Jeken Rico, J. Viquerat, A. Larcher, R. Valette, A. F. Sanches, V. Lannelongue, H. Ghraieb, R. Nemer, Y. Ozpeynirci, T. Liebig

**Affiliations:** 1 MINES Paris, PSL Research University, Centre de mise en forme des matériaux (CEMEF), CNRS UMR 7635, 06904 Sophia Antipolis Cedex, France; 2 Department of Neuroradiology, University Hospital Munich (LMU), Munich, Germany

**Keywords:** Learning algorithms, Biomedical engineering

## Abstract

Developing new capabilities to predict the risk of intracranial aneurysm rupture and to improve treatment outcomes in the follow-up of endovascular repair is of tremendous medical and societal interest, both to support decision-making and assessment of treatment options by medical doctors, and to improve the life quality and expectancy of patients. This study aims at identifying and characterizing novel flow-deviator stent devices through a high-fidelity computational framework that combines state-of-the-art numerical methods to accurately describe the mechanical exchanges between the blood flow, the aneurysm, and the flow-deviator and deep reinforcement learning algorithms to identify a new stent concepts enabling patient-specific treatment via accurate adjustment of the functional parameters in the implanted state.

## Introduction

An estimated 2–3% of the population harbour intracranial aneurysms (IAs)^1,2^, a pathological, localized sac-like outpouching of the arterial wall, whose rupture is the leading cause of nontraumatic subarachnoid haemorrhage, associated with a high rate of morbidity and mortality and a significant economic burden^3^. The increased frequency at which unruptured IAs are being diagnosed, due to the widespread use of cross-sectional neuroimaging in routine clinical practice, poses a persistent dilemma for physicians. This is imputable to the lack of definitive guidelines for optimal management, which is due to the high prevalence of aneurysms along with low rupture rates (with the annual occurrence of subarachnoid haemorrhage being about 10 per 100.000 persons^4^), and preventive treatment carrying risks of adverse complications^5^.

Aberrant vascular remodelling occurring through abnormal hemodynamic stress on blood vessels is believed to be a major factor in intracranial aneurysms pathophysiology, i.e., formation, growth, and stabilization or rupture^6^. The stress distribution, as determined by the blood flow and aneurysm geometry, elicits vascular remodelling via cell-mediated biologic pathways. This modifies the geometry, the stress, and drives further biologic processes, with rupture occurring when the stress on the aneurysm wall exceeds the yield strength of the material^7,8^. This biomechanical approach has proven relevant in assessing rupture risk, with hemodynamic indices such as flow-induced pressure (the stress normal to the vascular wall) and wall shear stress (WSS, the viscous frictional force exerted parallel to the blood flow) identified as potentially significant determinants of aneurysm natural history^9–12^.

Preventive treatment of unruptured intracranial aneurysms consists in occluding the sac to prevent blood from flowing directly into the aneurysm, which in turn helps reduce the stress on its wall. The two main options have long been surgical clipping and coiling^13^. Clipping is invasive, as it requires performing a craniotomy and exposing the aneurysm before placing surgical clips across the neck. While highly effective, clipping is constrained to easily accessible aneurysms and operations generally bear a substantial complication risk. Endovascular procedures, such as stenting and coiling, minimize the operational risk by avoiding open skull surgery. The latter approach involves filling the aneurysm sac with flexible platinum wires that dampen out ingoing blood jets and contribute towards the occlusion of the bulge. Since the wires are contained by the sac, wide neck IA or fusiform IA cannot be treated this way, due to the high risk of embolic disease and coil detachment.

In recent years, the implantation of flow-diverter (FD) stents has gained increasing acceptance among the interventional and neurosurgical communities as an effective alternative treatment option^14^. Such an approach consists in the endovascular deployment of flexible, highly conforming braided mesh devices along the parent artery and across the neck. The blood flow into the aneurysm is damped and redirected by the low porosity layer of FD wires covering the neck, reducing the overall circulation in the sac. The blood stagnation that follows a successful deployment causes a thrombus formation in the aneurysm cavity and a subsequent endothelialization of the neck^15^. In some cases, the completely occluded aneurysm is progressively reabsorbed by the parent vessel, precluding regrowth by hemodynamic mechanisms. Flow diverter stents have started a breakthrough in the endovascular management of intracranial aneurysms (including many wide-necked and fusiform aneurysms that were previously considered untreatable) but their mechanism of action is not thoroughly understood, as about 5–25% of aneurysms remain with circulation even after multiple-layer implantations^16^.

A substantial body of work is ongoing to improve aneurysm treatment outcomes by increasing the flow-diversion effect of the implanted stent^17,18^. The functional performance is largely dependent on implantation (e.g., sizing, landing zone) and geometrical features (e.g., braid angle, wire density, wire diameter) with wire material properties also being an important contributor. Hemodynamically, we believe that the pore density is a key parameter, as it must be high enough to occlude the aneurysm sac satisfactorily^15^, but not so high that it would trigger inflammatory remodelling associated with low-WSS values^19^. Nonetheless, there is currently a lack of empirical evidence supporting the superiority of one design over the others, meaning that the type of stent used for each patient is often based on the length of the lesion and the personal preference of the physician (even availability of stock). Therefore, the ability to design novel stent concepts from fast and accurate identification of patient-specific functional parameters is of utmost importance to provide clinical insight, optimize treatment decision-making, and improve prognosis. This has never been done before.

In order to make progress towards this objective, the present study combines multi-physics computational fluid dynamics (CFD) and deep reinforcement learning (DRL) to prove the applicability of such an optimization workflow for patient-specific stent design. On the one hand, CFD has risen to a prominent position in the endovascular research community due to its potential for rupture risk prediction via objective, quantitative, and mechanism-based parameters^20,21^, and its contribution to the design, development and evaluation of endovascular management methods^22,23^. On the other hand, DRL has been shown to perform with unprecedented efficiency in several areas, e.g., language processing^24^, robotics^25,26^, autonomous driving^27^, finance^28^ or healthcare management^29,30^, including recent inroads in computational biomechanics^31^.

The efforts for coupling CFD and DRL are developing rapidly, with a handful of pioneering studies providing insight into the performance improvements to be delivered in shape optimization^32–34^ and flow control^35–37^; see^38^ for a review. This is largely ascribed to the sustained efforts and commitment of the machine learning community, which has allowed expanding the scope from computationally inexpensive, low-dimensional model reductions^39–41^ to complex two- and three-dimensional Navier–Stokes systems^42–50^. Nonetheless, DRL has never been applied to hemodynamics computations (let alone biomedical flow computations in patient-specific geometries), even though we believe the field has matured up to the point where a breakthrough may be in reach for targeted control of unruptured intracranial aneurysms.

**Figure 1:**
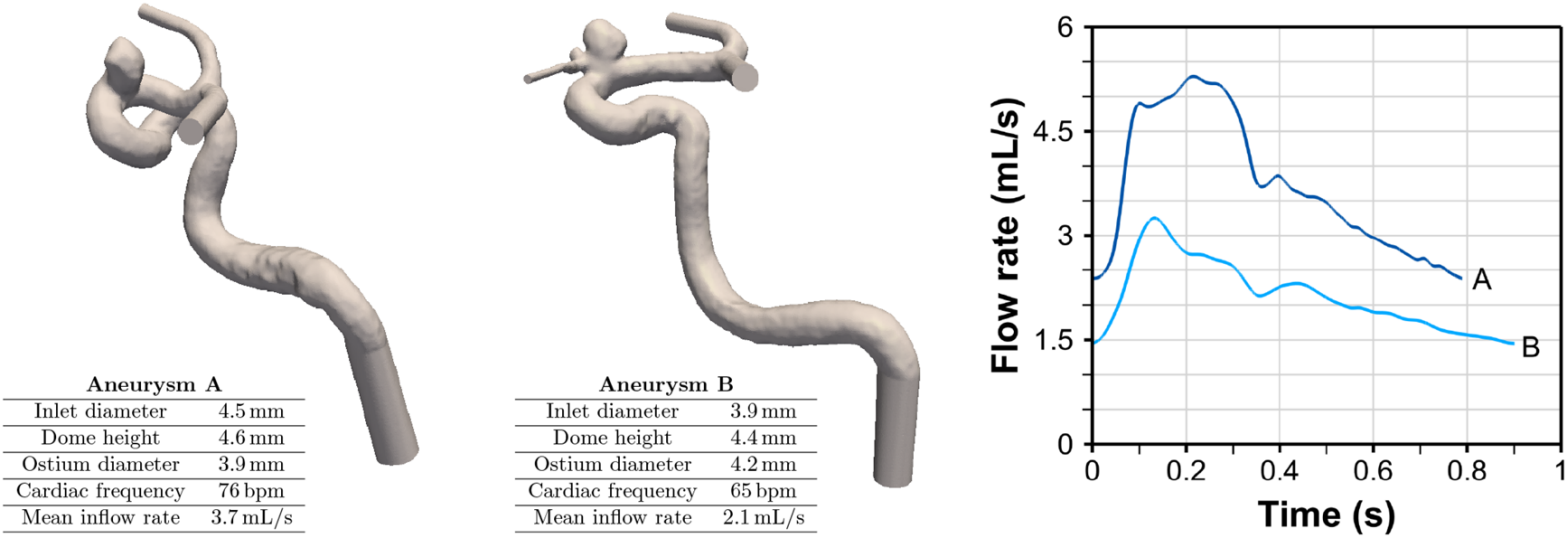
Patient-specific geometries and datasets for the two ICA aneurysms investigated in this study. The rightmost plot provides one period of the patient-specific inflow pulses used for the CFD simulations. The range of Reynolds numbers in the parent vessel (based on the inlet diameter, inlet velocity and infinite-shear rate viscosity) is 220-490 for patient A, and 160-370 for patient B (resp. at diastole and systole).

## Results

### Pre-stent hemodynamics

 Direct numerical simulations of the three-dimensional, incompressible Navier–Stokes equations performed with the Carreau–Yasuda rheological model of blood are used to investigate two patient-specific models of unruptured intracranial aneurysm. In the first place, the focus is drawn on the geometry labelled A, whose vascular information is provided in Fig. 1. It is a side-wall, wide-neck aneurysm of the supraclinoid internal carotid artery (ICA), proximal to the ICA bifurcation into the anterior cerebral artery (ACA) and the middle cerebral artery (MCA); see Fig. 1 for provision of the detailed vascular information. The posterior communicating artery (PComA) is neglected, as it branches off well past the aneurysm. The ophthalmic artery (OA) is also neglected, although it branches off at this section of the ICA, in the vicinity of the aneurysm. Nonetheless, we do not anticipate any significant effect on the hemodynamics given its patient-specific smallness ($\sim 0.5$ mm in diameter), plus this outflow is often neglected in numerical simulations as it is a common clinical practice to let flow diverters occlude it if no other viable option is present^51^. The simplified model therefore ultimately features a single source of inflow (the ICA) and two outflows (ACA/MCA).

**Figure 2:**
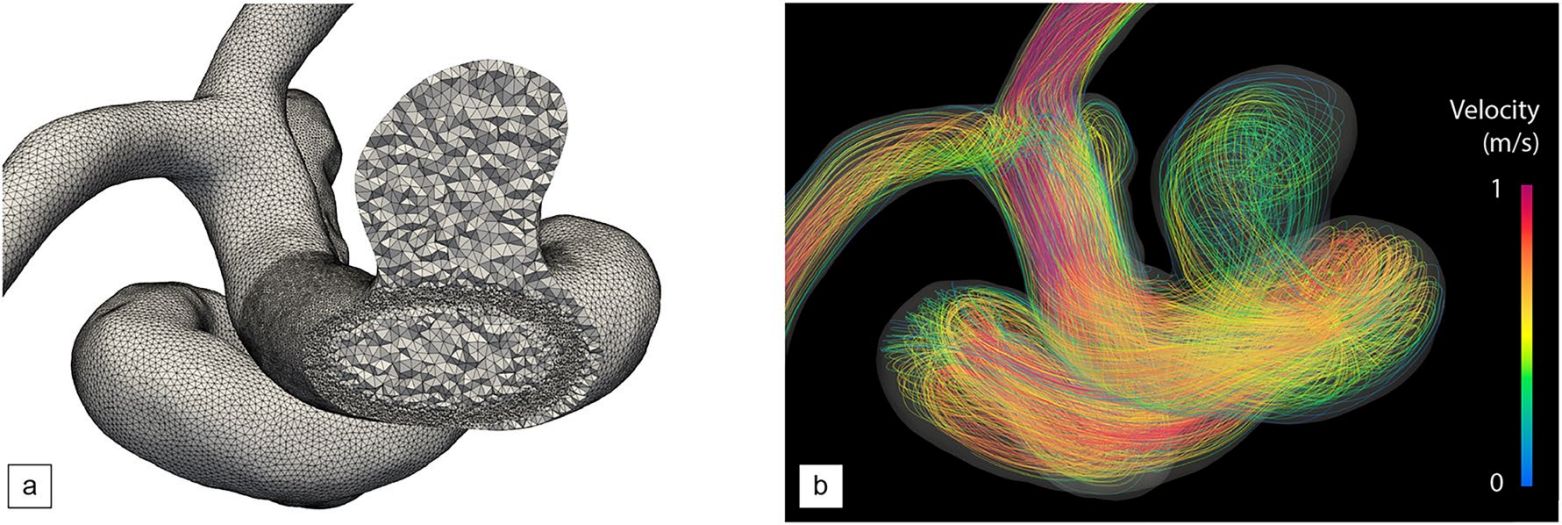
Pre-operative hemodynamics of aneurysm A. (**a**) Cut view of the employed anisotropic tetrahedral mesh. (**b**) Velocity streamlines at systole.

**Figure 3:**
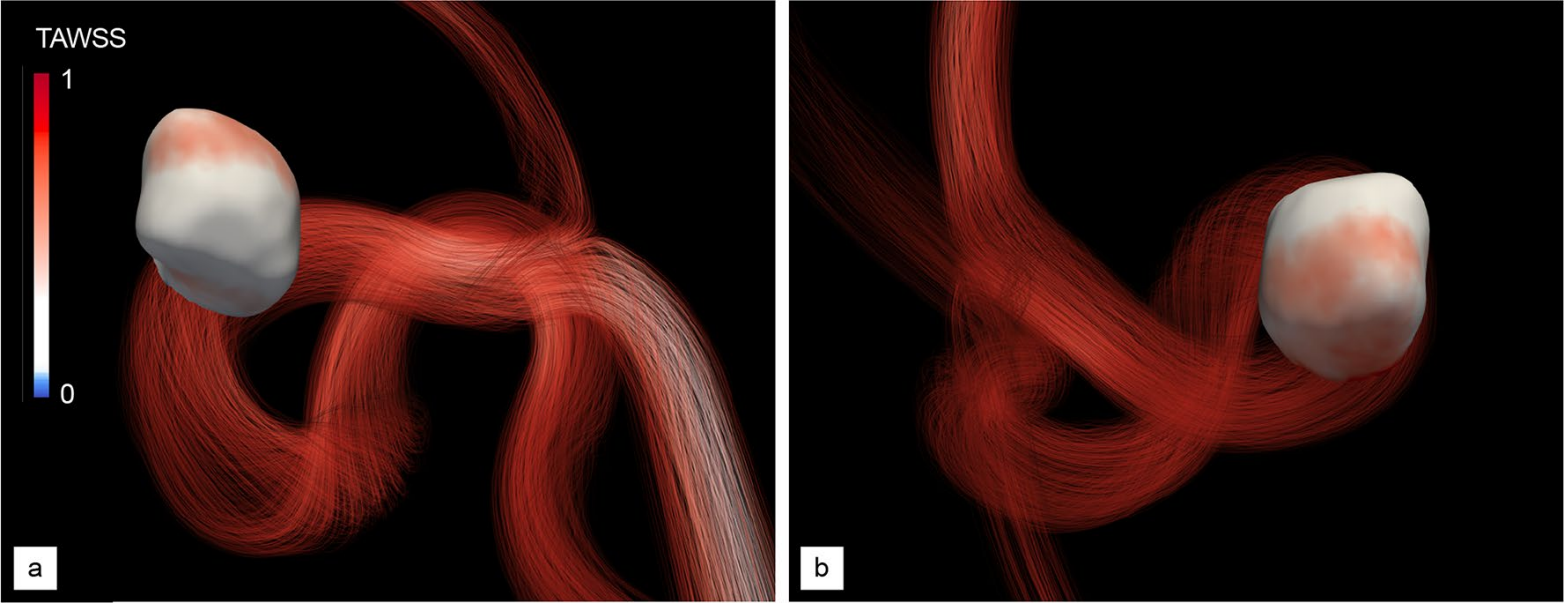
Pre-operative normalized TAWSS of aneurysm A. (**a**) Proximal view. (**b**) Distal view. The color scale has been adjusted to emphasize low/high WSS areas associated with remodelling and rupture risk^52^.

The vessel walls, taken to be impermeable and rigid, are treated with body-fitted, unstructured adapted grids (see Fig. 2a). The numerical solutions are customized to the patient (also labelled A) specific physiology using vascular geometries reconstructed from three-dimensional rotational angiography (3D-RA) images and pulsatile volumetric inflow rates adjusted to two-dimensional phase-contrast magnetic resonance imaging (2D-PCMRI) measurements. All quantities of interest, including velocity, WSS (the local, instantaneous, intra-saccular wall shear stress of pivotal importance in this context), SAWSS (the instantaneous WSS spatially averaged over all intra-saccular positions), and TAWSS (the local WSS averaged over a cardiac cycle), are computed from parallel hemodynamics simulations run over two cardiac cycles (representing approximately 1.6 s of physical time) with a reference inflow sequence of consecutive pulses starting in the end-diastolic state. This is because the solution has settled into regular, sinusoidal oscillations by the end of the first cycle, as obtained from preliminary comparison of WSS data over up to ten cardiac cycle.

The peak-systolic streamlines in Figs. 2 and 3 show that the flow remains close to parabolic in the inflow segment (hence reminiscent of Hagen–Poiseuille flow), but quickly becomes helical because the curved vessel geometry acts as a source of flow instability, as recently assessed in patient-specific geometries^53^. Most of the blood enters the aneurysm at the proximal part of the neck in the form of a high-speed jet ($\sim$ 0.73 m/s in velocity magnitude), impinging on and reflecting off the aneurysm wall, rolling up into complex vortical structures and finally swirling out of the bulge and to the outflow segments. This creates a strongly heterogeneous WSS pattern, with most of the distal part of the dome sustaining high WSS classically associated with aneurysm growth and rupture, as clearly illustrated in Fig. 3a, b. Maximum local, instantaneous WSS values of more than $\sim$ 169 dyne/cm$^{2}$  have been measured near the impaction zone, which is about 8 times normal WSS in cerebral arteries^52^.

### Virtual stenting

 Endovascular treatment is modeled by wrapping a distribution of identical, cylindrical wires around a toroidal envelope inscribed in the arterial segment containing the aneurysm (half of them clockwise and the other counter-clockwise). In order to achieve heterogeneous functional parameters (in the sense that the pre-deployment stent structure must have variable pore density and porosity), the proximal end section of the envelope is divided into four quadrants, each of which with a specific (possibly different) number of uniformly distributed wires.

The parametrization foresees the modification of six design variables: the number of wires in each group, their radius and a winding factor (the same for all wires) that controls the local braiding angle between wires. Given the difference in scales between the vascular vessels (about a few mm in diameter) and the stent strut thickness (about a few ten $\upmu$m), we rely on a hybrid meshing approach wherein the stents are embedded in the body-fitted vascular grid^54,55^. Anisotropic adaptation in the vicinity of the stent envelope, as shown in Fig. 2a. We then use the monolithic immersed volume method (IVM^56^) together with anisotropic mesh adaptation in the vicinity of the stent envelope to solve the interaction between the blood flow and the stent material. This allows easy handling of any complex device whose struts may be in contact with, or form very small gaps with the vessel walls, without additionally conforming the vascular grid to the stent geometry.

**Figure 4:**
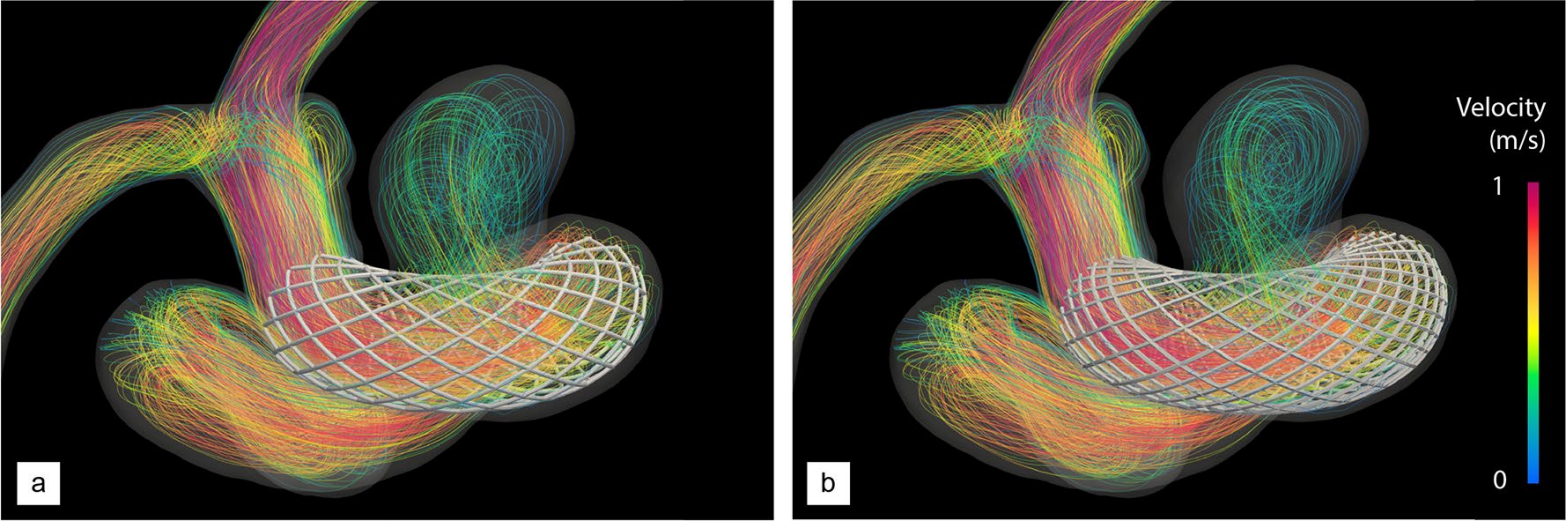
Post-operative, peak-systolic velocity streamlines of aneurysm A. (**a**) After treatment with a homogeneous stent made of 24 wires. (**b**) After treatment with a 34 wire, non-uniformly braided stent. The stent design is the result of the optimization provided in the following sections (see DRL optimization).

The proposed approach is first used to assess the ability of the method to capture numerically the flow diversion effect using a standard homogeneous stent strut distribution made of 24 wires (12 in each braiding direction, 3 in each group) with a radius set to 60 µm. We believe this is a reasonable compromise between desirability and feasibility, as thinner wires mirroring more accurately those of real medical stents (whose radii are in a range from about 15 to 30 µm) would escalate the CPU time and memory requirements (due to the need to embed large stent meshes and to additionally refine the vascular grids). All wires are braided with winding factor 25, that yields a braiding angle of 75$^\circ$ and a porosity of about 68.5%, (pore density of 3.1 pores/mm^2^), all values estimated from a pre-deployment, cylindrical stent structure.

**Figure 5:**
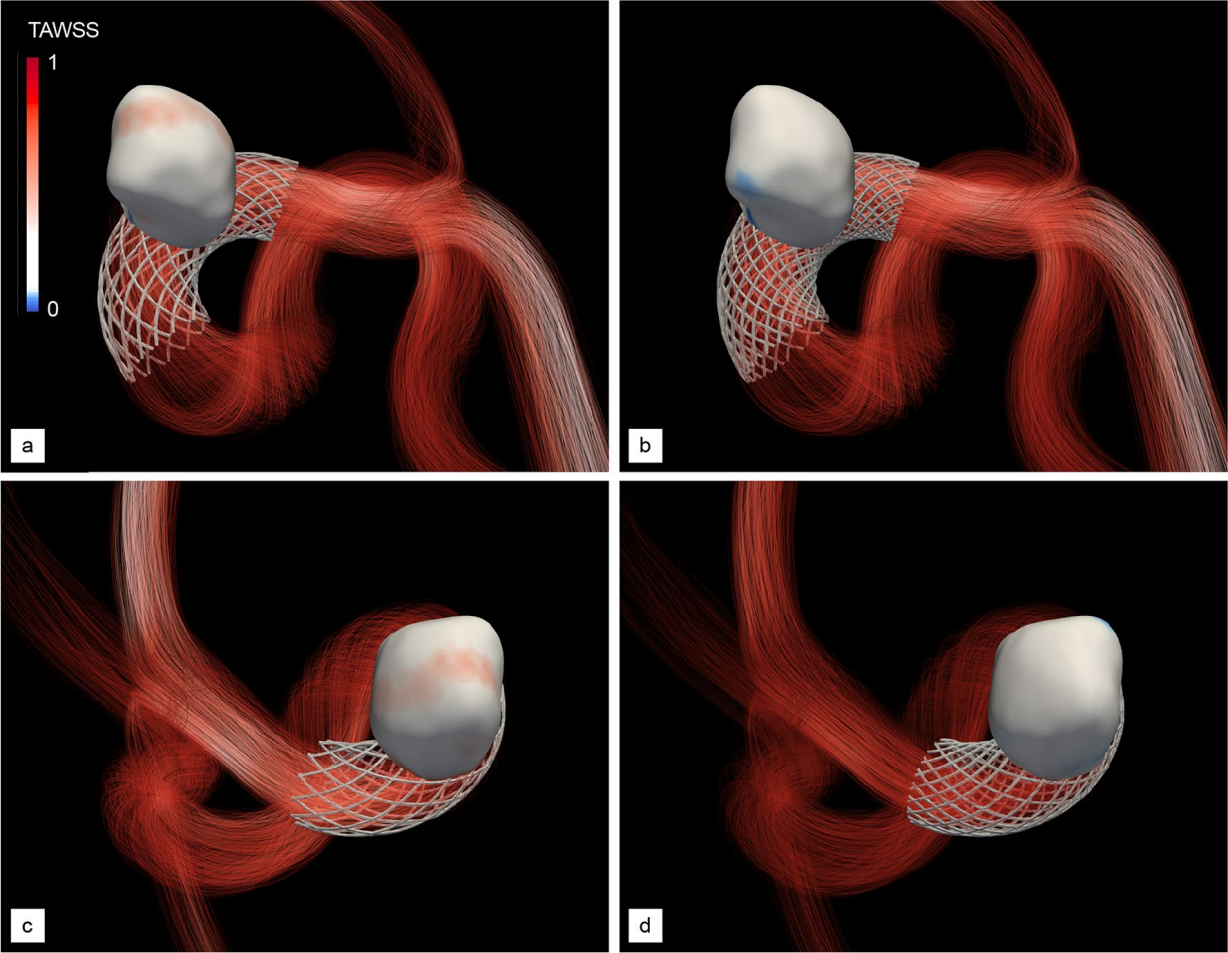
Post-operative, normalized TAWSS of aneurysm A. (**a**, **c**) Proximal and distal views after treatment with a homogeneous stent made of 24 wires. (**b**, **d**) Same as (**a**, **c**) after treatment with the optimal, non-homogeneous stent provided by our DRL framework, made of 34 non-uniformly braided wires (see Fig. 6d). Values are normalized based on the maximum TAWSS encountered in the pre-operative configuration (see Fig. 3).

Visualizations of the inflow jet, intra-saccular flow pattern (Fig. 4a) show that the stent significantly disrupts the blood flow, more of which is diverted away from the aneurysm and flows directly to the outflow segments. Nonetheless, the flow organization inside the aneurysm is essentially reminiscent of its unstented counterpart, with blood entering at the proximal part of the neck and swirling in and out after impinging on the distal wall. The key difference lies in the inflow jet having lower velocity (about 0.60 m/s in velocity magnitude) and weaker shear, hence less vorticity, and ultimately less shear stress on the aneurysm wall. This is further illustrated by the TAWSS distributions in Fig. 5a, c, where the instantaneous WSS peaks at about $\sim$ 120 dyne/cm$^{2}$ . This represents a reduction of about 30% with respect to the unstented case illustrated in Fig. 3, although we notice the persistence of a heterogeneous WSS pattern over the distal part of the dome.

### DRL optimization

 The optimization objective considered herein consists of bringing back the post-operative value of MWSS (defined as the maximum of SAWSS over a full cardiac cycle) to a setpoint of half the pre-operative value, hence the reward

1 $$\begin{aligned} r&= -\left| \text {MWSS} - \text {MWSS}_{\text {ref}} \right| \qquad \text {with}\qquad \text {MWSS}_{\text {ref}}=\frac{\text {MWSS}_{\text {0}}}{2}\,, \end{aligned}$$

where the 0 subscript denotes a pre-stent quantity. This choice is intended to reduce high WSS associated with aneurysm growth and rupture, while avoiding low WSS conditions that might initiate apoptotic pathways via undesired vascular remodelling^19^. In practice, the MWSS_0_ for patient A is 76.6 dyne/cm$^{2}$  (roughly half the maximum local, instantaneous value reported above), which yields a setpoint of 38.8 dyne/cm$^{2}$ .

**Figure 6:**
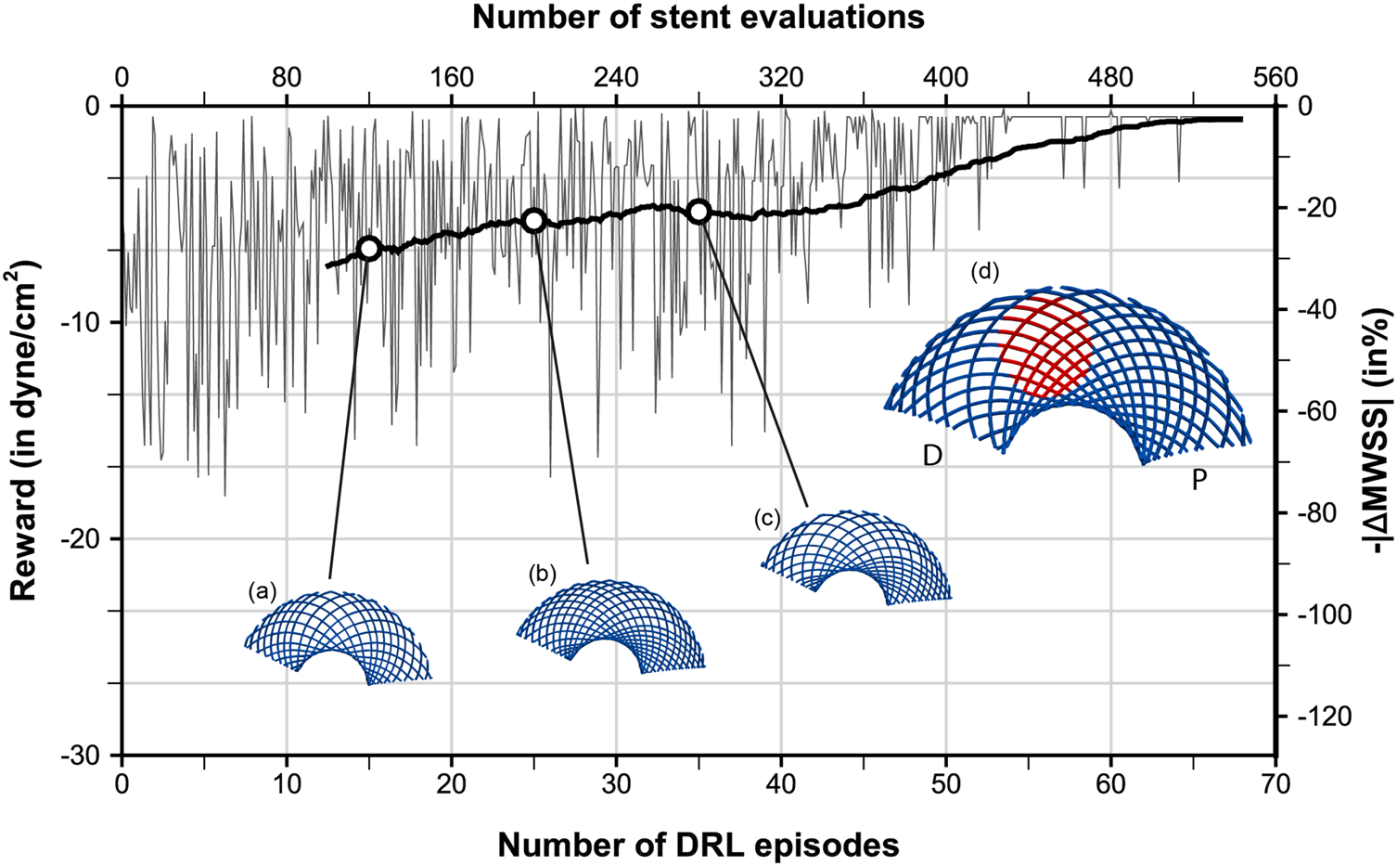
Stent optimization along with deep reinforcement learning for aneurysm A. The fine line represents the evolution per episode of the instant reward, while the thick line is the moving average reward computed over the 100 latest values. The right vertical axis presents the relative variations of MWSS with respect to the setpoint of half the pre-operative value. Representative stents generated over the course of optimization are superimposed, with the stent generated at episodes 15, 25 and 35 shown in (**a**–**c**) and the optimal stent predominantly generated after episode 55 shown in (**d**). The P/D labels indicate the proximal and distal end sections of the stent, whose upper half is displayed from below (longitudinal cut) to single out the region covering the aneurysm neck (shown by the red patch in (**d**)).

Single-step PPO, a reinforcement algorithm intended for situations where the optimal policy is independent of state^48^, is used to evolve five (out the six) design parameters, as the wire radius has been set to 60 µm to keep the computational cost affordable, although it is a free parameter that could also be learnt from data. For each learning episode, the DRL agent (a deep neural network) therefore outputs five discrete values: four values $\{n_{j\in 1\dots 4}\}$ between 3 and 7 in step of 1 for the numbers of wires (one per set of equally spaced wires) and one value *k* between 20 and 35 in steps of 5 for the winding factor, hence 2500 parameter combinations comprising between 24 and 56 wires in total. The generated stent configurations have nominal braiding angles in a range from 65$^\circ$ to 95$^\circ$ and porosities in a range from 30.5 to 68.5% (pore densities between 3.1 and 22.7 pores/mm^2^). all values estimated from pre-deployment, cylindrical stent structures. The reward evaluation proceeds from hemodynamics simulations run over two cardiac cycles, with MWSS computed over the second cycle, after which the network is updated for 32 epochs using 8 environments and 2 steps mini-batches.

A total of 68 episodes have been run for this case, which represents 544 simulations, each of which lasts 20 min using 32 cores, hence 5,760 h of total CPU cost (equivalently, 45 h of resolution time). A moving average reward is also computed as the sliding average over the 100 latest values to assess convergence a posteriori (see Fig. 6). The reward convergence history in Fig. 6 evidences the successful convergence of the PPO algorithm coupled with patient-specific hemodynamic simulations. After 50 episodes (representing 400 simulations, hence 400 out of the 2500 possible designs), the DRL agent indeed starts to systematically pick the specific stent shown in Fig. 6d, whose red patch singles out the region of interest located in front of the aneurysm neck. The latter is made of 34 wires (17 in each braiding direction, distributed into four groups of 5, 3, 5 and 4 wires, respectively) braided with winding factor 25. This yields in a nominal average porosity of 55%, with pore densities (in deployed state) ranging between 2.3 and 16.0/mm^2^ in the neck region facing the aneurysm.

Such a design is meant to be optimal for the patient specific aneurysm geometry and pulse, which is assessed now by comparing numerically the post-operative hemodynamics treated with the optimal stent designed by DRL (that earns a MWSS of 37.8 dyne/cm$^{2}$  differing from the intended setpoint by 1%) and with the standard homogeneous stent considered so far (that earns a MWSS of 54.7 and is clearly inferior for the chosen reward). The peak-systolic streamlines in Fig. 4 illustrates the different flow deviation effect of the optimal stent. The latter successfully and adequately cuts down the inflow jet (that was inducing high WSS values on the distal part of the bulge, whose velocity magnitude is now about 0.47 m/s) while substantially altering the intra-saccular flow organization, found to involve fewer vortex structures, more parallel streamlines, and less swirling. We note that the optimal stent also substantially reduces the blood velocity at the exit of the aneurysm: 0.26 m/s, to be compared to 0.35 m/s without stent and using the standard, homogeneous stent. The result on the WSS distribution is even more patent, as Fig. 5 shows that the DRL stent has completely eliminated the unstented area of maximum WSS (the local, instantaneous WSS now peaks at about 91 dyne/cm$^{2}$ , which represents a reduction of about 45% with respect to the unstented case.) while restoring an almost homogeneous WSS pattern, which the homogeneous stent had failed to achieve.

### Generalizability study

 For the sake of generalization (and in order to assess suitability for various aneurysm configurations), we apply now the DRL framework to a second patient-specific model of untreated, unruptured intracranial aneurysm (labelled B) whose vascular information is provided in Fig. 1. It is a saccular, multilobulated aneurysm located on the ICA, at the junction with the ophthalmic artery (OA). The latter is thus retained in the model (as it cannot be cleanly removed), yet not occluded numerically, as we voluntarily let blood flow from the ICA into the OA across the stent to explore the model ability to handle more complex intra-aneurysmal flow conditions.

**Figure 7:**
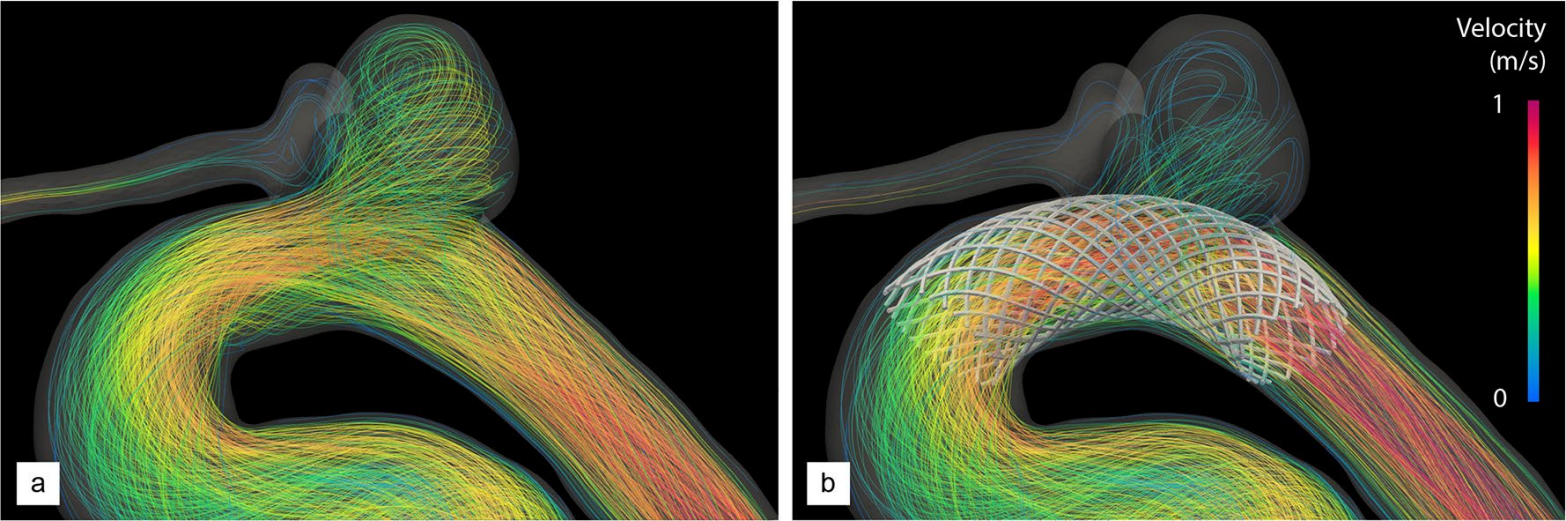
Velocity streamlines of aneurysm B without and with DRL-optimized stent. (**a**) Pre-operative state. (**b**) Post-operative flow after insertion of the optimal stent (see Fig. 9d).

**Figure 8:**
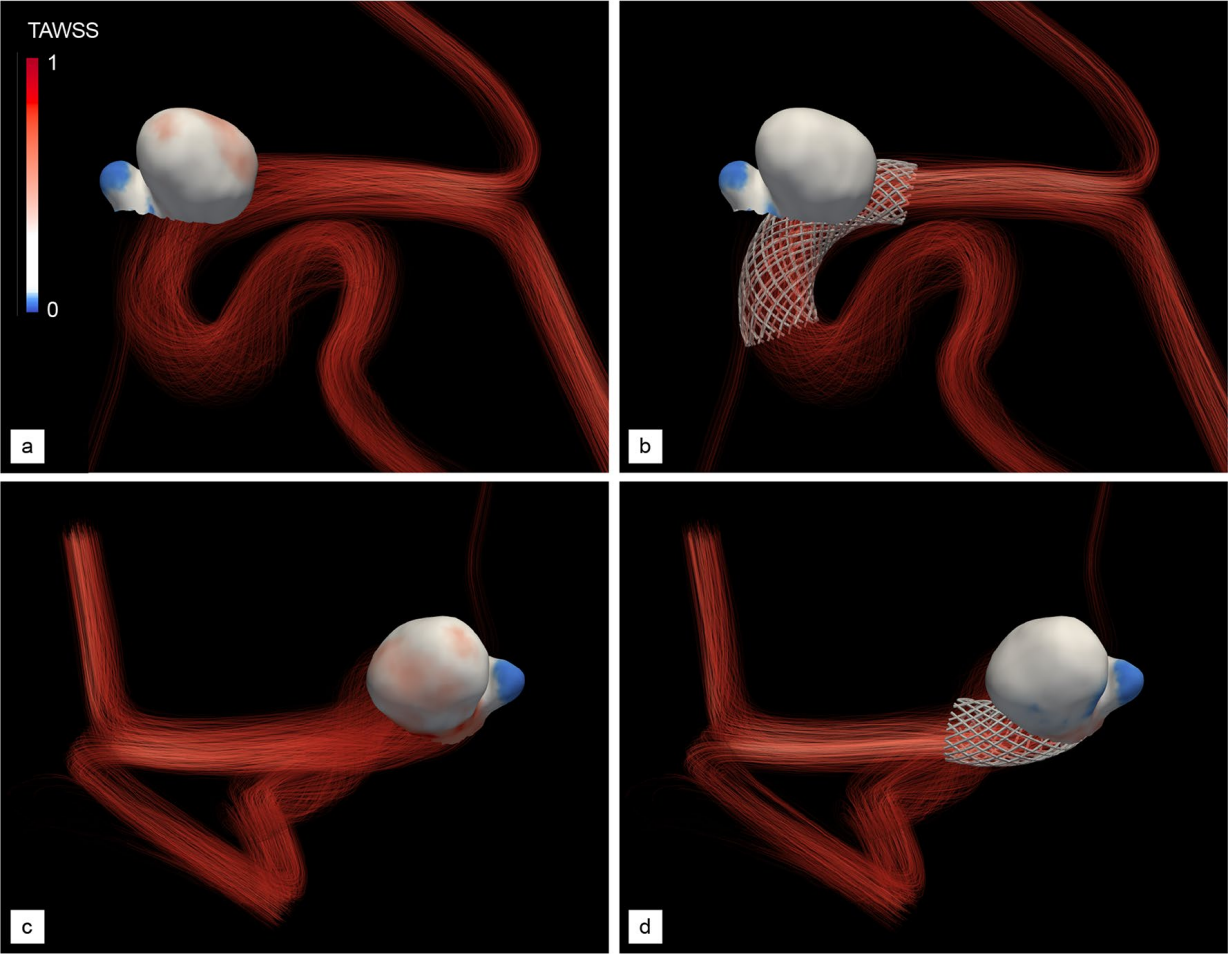
Normalized TAWSS of aneurysm B without and with DRL-optimized stent. (**a**, **c**) Proximal and distal views in the pre-operative state. (**b**, **d**) Same as (**a**, **c**) after treatment with the optimal, non-homogeneous stent provided by our DRL framework, made of 30 non-uniformly braided wires (see Fig. 9d). Values are normalized based on the maximum TAWSS encountered in the pre-operative configuration (see Fig. 3).

Following the same steps as for the previous case, the pre-operative hemodynamics of this patient has been analyzed from numerical simulations customized to his/her physiology (both in terms of vascular geometry and inflow pulse) carried out over two cardiac cycles (representing 1.8 s of physical time). The peak-systolic streamlines shown in Fig. 7a show that the case has many similarities to that of patient A, as the flow quickly becomes helical, and most of the blood enters the aneurysm at the proximal part of the neck in the form of a high-speed jet (0.72 m/s in velocity magnitude), that traverses the entirety of the primary bulge, impinges on its distal wall, rolls up into complex vortical structures and finally swirls out to the outflow segments (including the OA). As illustrated in Fig. 8a, c, this again yields a strongly heterogeneous WSS pattern, with high WSS values up to $\sim$ 146 dyne/cm$^{2}$  in the vicinity of the neck and in most of the distal part of the dome, but low WSS in the daughter sac, that turns to be barely exposed to the blood flow environment.

**Figure 9:**
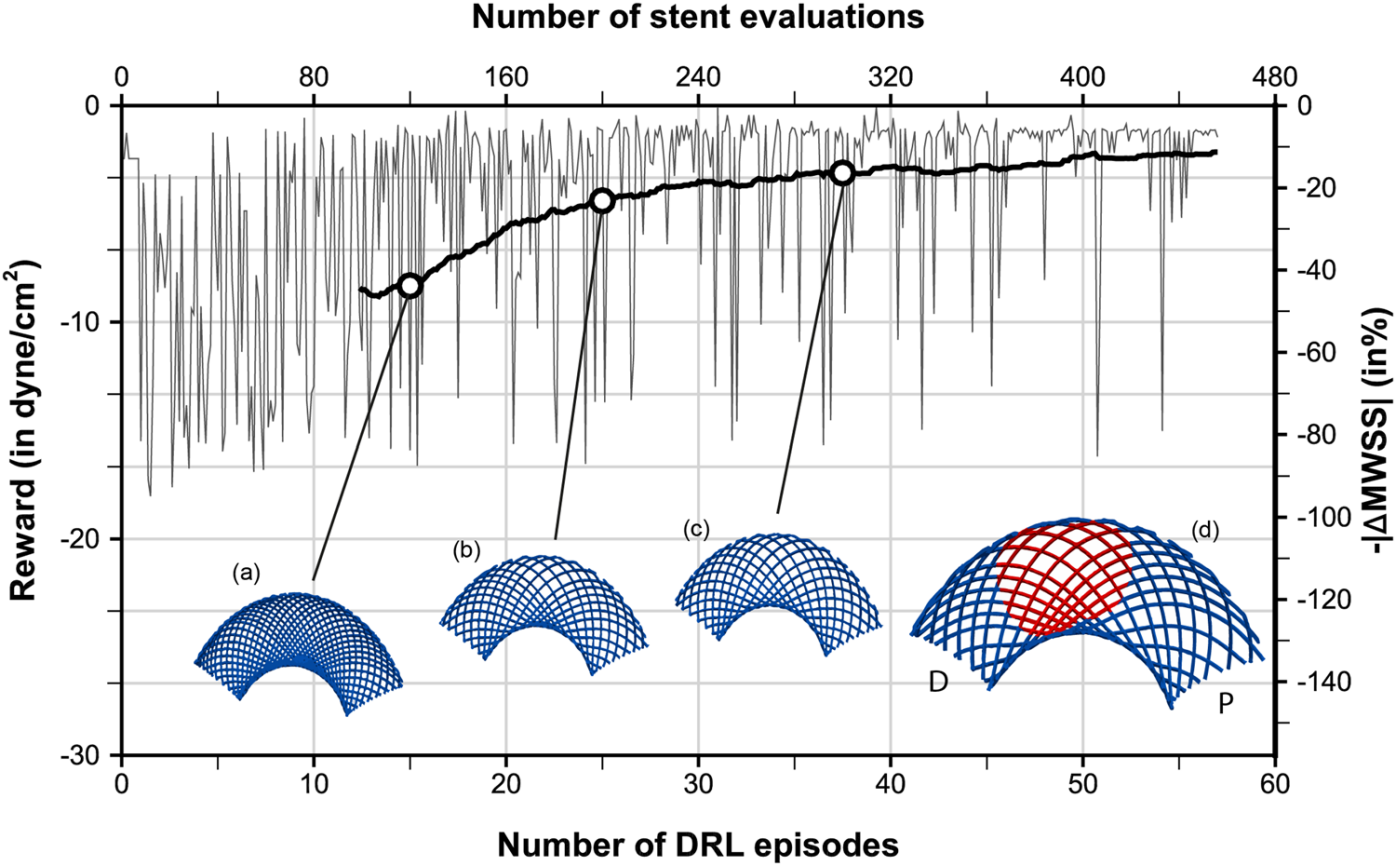
Stent optimization along deep reinforcement learning for aneurysm B. The fine line represents the evolution per episode of the instant reward, while the thick line is the moving average reward computed over the 100 latest values. The right vertical axis presents the relative variations of MWSS with respect to the setpoint of half the pre-operative value. Representative stents generated over the course of optimization are superimposed, with the stent generated at episodes 15, 25 and 35 shown in (**a**–**c**) and the optimal stent predominantly generated after episode 45 shown in (**d**). P and D annotations indicate the proximal and distal end sections of the stent, respectively.

The DRL optimization has then been run in similar fashion over a total of 58 episodes (464 simulations) using a setpoint of 36.3 dyne/cm$^{2}$  (MWSS_0_ for this patient is 72.6 dyne/cm$^{2}$ ,) and evaluated after convergence (accounted for when the agent outputs a majority of one specific design over several episodes). The reward convergence history in . 9 evidences good convergence after 45 episodes (representing 360 simulations, hence 360 out of the 2500 possible designs). The DRL agent then starts to sample the specific stent shown in Fig. 9d, whose red patch singles out the region of interest located in front of the aneurysm neck. The latter earns a MWSS value is 37.2 dyne/cm$^{2}$ , which differs from the intended setpoint by 3%. It is made of 30 wires (15 in each braiding direction, distributed into four groups of 5, 3, 4 and 3 wires, respectively) braided with winding factor 25. This yields in a nominal average porosity of 61%, with pore densities (in deployed state) ranging between 4.1 and 11.2/mm^2^ in the neck region facing the aneurysm. We note that the convergence is slightly less good than for patient A, which may be because the neck of aneurysm B is larger, but the same ranges of design parameters have been used for both patients. This offers more leeway by allowing more threads to fit into the region of interest of patient B, which, combined to the fact that only a small number of discrete winding factors are evaluated, increases the sharpness of the reward, known to be detrimental to the conservative policy updates of the PPO algorithm^48^.

Finally, the efficiency of this design (in fact different from the optimal determined for patient A) has been assessed by comparison of the pre- and post-operative hemodynamics computed after treatment with the optimal stent. The peak-systolic streamlines in Fig. 7b show that the blood flow is adequately diverted away from the neck and into the parent vessel. The blood velocity is reduced at the entry (0.38 m/s in magnitude) but also at the exit of the aneurysm (0.23 m/s, to be compared to 0.43 m/s without stent), while swirling is essentially suppressed, as the inflow jet now merely slides along the distal wall. This considerably reduces the high distal values of WSS, as the maximum local, instantaneous WSS is now about 80 dyne/cm$^{2}$  (again a reduction by about 45% with respect to the unstented case). We also note an almost homogeneous WSS pattern is restored in Fig.  8b, d, save for the low WSS region in the daughter sac, meaning that the latter could still grow on its own via inflammatory and apoptotic remodelling.

## Discussion

### Optimal stents feature a gradient of porosity

 Reported results highlight the potential of DRL shape optimization for endovascular stenting of intracranial aneurysms. It should be emphasized the optimal designed generated by our DRL agent, all relative to the chosen reward functions, feature varying porosities in the region of interest (ROI) facing the aneurysm neck. This, by itself, could represent a breakthrough in the stent manufacturing industry, where such designs do not yet exist (to date, variable porosity can be achieved locally only by superposition of several flow-diverters, which increases the risk and the cost of the surgery). More importantly, the optimal porosity gradient differs for both aneurysms, which paves the way for developing novel devices tailored to the patient specific aneurysm, including (but not limited to) its geometry and pulse.

The results show that the optimal stents successfully cut down the blood velocity at the entry and the exit of the aneurysm while also altering the swirling flow inside the aneurysm, either by subtly modifying the swirling direction (patient A), or by suppressing swirling altogether (patient B). Nonetheless, the complexity of the correlation between local porosity distribution, flow deviation and hemodynamics makes it difficult to unravel the exact physical mechanism behind the efficiency of this or that design, although it is common knowledge that the stent must allow blood in and out to avoid the occurrence of too-low WSS values, while sufficiently impeding the blood flow associated with the highest values of WSS in the aneurysm. From this perspective, the DRL approach is beneficial in two important respects: first, it is efficient, even though the parameter spaces are large and it may be costly to identify optimal designs from simple parametric searches. Second, and more significantly, it succeeds in discovering optimal designs from unforeseen parameter combinations, without any priori knowledge or assumptions about hemodynamics concepts.

### CFD modelling assumptions and limitations

 Computational blood flow modelling in intracranial aneurysms has tremendous potential, yet limited applicability in a clinical context because of the simplifying assumptions that are traditionally (and often implicitly) made^57,58^. Chief among them is the fact that walls are almost always assumed rigid, while arteries are compliant vessels, i.e., they deform under the shear stress of blood flow, with possibly large displacements impacting the WSS estimates (the authors in^59^ report 10-30 % WSS reductions compared to rigid wall simulations). A two-way coupled fluid-structure interaction (FSI) analysis is thus necessary to solve accurately the mechanical exchanges between the blood flow and the arterial tissue, while also encompassing the stent deformation occurring under load conditions (by the blood flow and/or the arterial tissue), another important factor that may alter the porosity at the neck and impair the long-term efficiency^60,61^. One ongoing debate regarding the need to include the effects of compliance comes down to whether the uncertainties or inaccuracies in the data needed to model its effects may mask any perceived benefit of doing so: on the one hand, it has been acknowledged that improved computational models should incorporate patient-specific, spatially varying wall thicknesses, as uniform wall properties and thicknesses based on literature values will fail to represent inter- and intra-individual variations^62,63^. On the other hand, it is feasible to measure individual wall properties by imaging and inverse modelling techniques, but such non-linear analyses introduce substantial uncertainties, for instance imaging can distort wall thickness measurements in a way that can be difficult to detect or correct^64^. In this regards, it is reasonable and expedient to use a rigid wall model for the present purpose of showcasing the use of DRL techniques for image-based CFD hemodynamics optimization (without any consideration of being directly applicable real medical cases), while leaving to future research to more fully address this issue and close the methodological gap of providing high-fidelity hemodynamic data. Finally, achieving the fine deployment of the stent in the arterial vessel was not in the scope of this study. A realistic deployment, along with a more versatile stent parametrization would expand the possible configurations and surely lead to fascinating results, which could drastically impact the medical community.

### DRL reward function

 There are two main aspects worth discussing regarding the DRL reward function used herein. First, the design of a feasible reward function is one of the challenges in reinforcement learning problems, but one that is barely discussed in the available literature. In the absence of best practice guidelines, it is essentially a trial-and-error exercise, with a human expert defining an initial reward function based on his/her knowledge of the problem, observing how the agent performs, then tweaking the reward function to achieve greater performance. We use here a reward function aligned with the objective function, meaning that when the agent is learning to maximise this reward, it is also learning to minimize the distance between the post-stent maximum value of MWSS over a cardiac cycle and the setpoint of half the pre-stent value. This allows reducing WSS while preventing the occurrence of very low WSS values, which is consistent with the expected outcome of a stenting operation (in the absence of further quantitative information or reduction objectives). A more sophisticated approach to pursue in future work could be to force the WSS to remain in a physiological range (that could be defined from patient-specific data) at every point in the bulge, using for instance a local reward function defined as

2 $$\begin{aligned} r = \int _S r_{loc}({\varvec{x}}) \,ds\quad \text{ with }\quad r_{loc}({\varvec{x}}) = {\left\{ \begin{array}{ll} \text {WSS}({\varvec{x}}) - \text {WSS}_{inf} &{} \text {if }\;\; \text {WSS}({\varvec{x}})< \text {WSS}_{inf},\\ 0 &{} \text {if }\;\; \text {WSS}_{inf} \le \text {WSS}({\varvec{x}}) \le \text {WSS}_{sup},\\ \text {WSS}_{sup} - \text {WSS}({\varvec{x}}) &{} \text {if }\;\; \text {WSS}_{sup} < \text {WSS}({\varvec{x}})\,. \end{array}\right. } \end{aligned}$$

Second, the reward uses MWSS as the sole predictor of aneurysm rupture, which implicitly assumes that a brief exposure to extended regions of high WSS is key towards predisposing the aneurysm wall to weakening and rupture. On the one hand, this suffices to lay the foundation for future research in this field, given the wide acceptance of WSS as a key factor in the physiological and pathological response of cerebral arteries. On the other hand, a gap of knowledge remains on this issue (for instance, both high or low WSS have been separately correlated to aneurysmal formation and growth^9,19,20,65,66^), and enriched reward functions (encompassing the time and space-dependent influence of blood dragging at the aneurysm wall, both in magnitude and in direction) are likely needed to improve clinical relevance. In this regards, it is worth insisting that the presented framework is highly generalizable, in the sense that it can assess new concepts of flow-deviator stents with respect to any or any combination of the markers of disturbed blood flow that have surfaced in recent publications (WSS gradient, oscillatory shear index, relative residence time, to name a few), that reflect different assumptions being made about the hemodynamic conditions driving the progression of intracranial aneurysms toward rupture^53,67–70^. This falls under the scope of multi-objective DRL for which there are two main approaches. The most common way is to use a linear function to transform the multi-objective problem into a standard single-objective problem. Another interesting (but very costly) strategy is to explicitly separate the individual components of the reward function, in order to better understand the policy trade-off (the related methods, based on the Pareto optimum, are not yet frequently applied to DRL problems).

### DRL algorithm

 Future work should aim at further improving the flexibility of the proposed framework by allowing more realistic stent geometries (in terms of wire radius, number of wires), thus increasing the number of possible stent designs. Having a more continuous optimization space (by increasing the number of winding factors) will also undoubtedly improve the convergence of the PPO algorithm. From this standpoint, it should to be emphasized that this is a proof-of-concept study and that convergence and efficiency (i.e., the number of stent designs that need to be evaluated to reach convergence) could be accelerated by hyper-parameter tuning or using pre-trained deep learning models (as is done for instance in transfer learning). Generally, the rather simplistic PPO framework could be substituted by a more elaborated algorithm, for instance Policy-based Optimization (PBO)^71^, another single-step reinforcement algorithm that samples actions from full covariance matrices, and is theoretically better suited to represent higher order logic and to handle complex parameter interactions.

### Future research directions

 The purpose of this study is to lay out the foundation for future research in this field. We anticipate that large-scale studies with long-term follow-up will allow developing more reliable risk-prediction models. As envisioned by Meng et al.^19^, we picture that intracranial aneurysms could be sorted into different categories associated with different predictors reflecting different growth and rupture mechanisms (say, high WSS and positive WSS gradient for narrow-necked aneurysms vs. low WSS and high fluctuations of WSS orientation for wide-necked aneurysms), at which point a high-fidelity DRL-CFD hemodynamics framework accurately modelling the elastic deformation of the parent artery will be instrumental in providing clinically relevant, patient-specific stent designs (except for the unpredictable delivery manipulations and variations of vessel geometry occurring during the intervention that still might impact the stent implantation). By then, it is reasonable to expect that further developments in the fast-moving field of deep reinforcement learning will allow for faster convergence and lesser execution load (using, e.g., auto-encoders and systematic state compression, or on-the-fly generation of surrogate models with uncertainty level prediction). This should set up a framework fast enough to inform design in a matter of hours rather than days, which in turn will reliably augment the current clinical diagnostics capabilities. Another reason to push DRL forward in this context is the ability of neural networks to transfer knowledge from previous experiences, to quickly adapt to different environments (i.e., different patient-speciffic numerical models of intracranial aneurysms, corresponding to new patients in practical applications) and effectively learn new tasks (i.e., different rewards, to achieve further refinement of risk prediction). We expect that this will be a key feature to reduce learning time and improved neural network performance, as progress are made towards realizing the clinical utility of CFD for assessment of intracranial aneurysm rupture.

## Methods

### Clinical and imaging data

 Images obtained from 3 Tesla MRI (magnetic resonance imaging) and 3D-DSA (digital subtractiob angiography) to create the adequate geometry for the simulation and the pulse to impose the flow. All images have been acquired at the University Hospital - LMU Munich.

### Stent model

**Figure 10:**
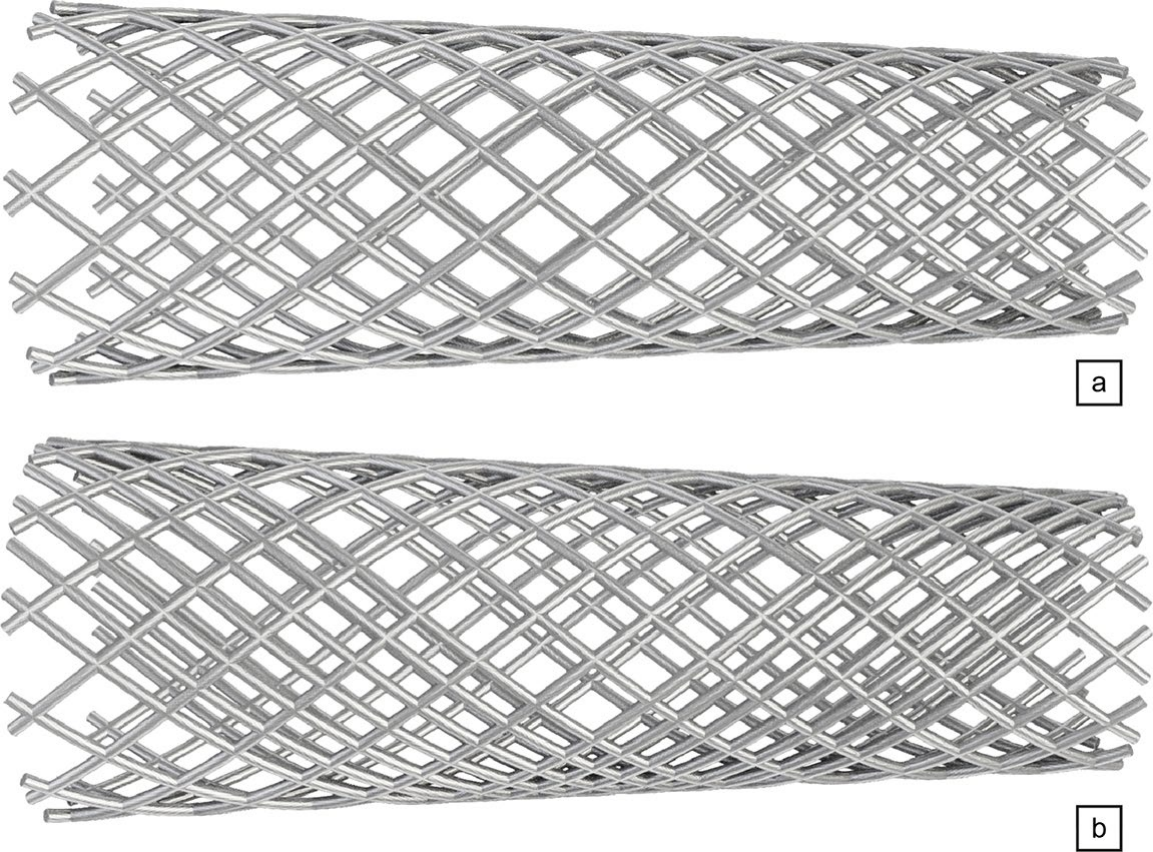
Stent generation examples. Homogeneous (**a**) and heterogeneous (**b**) braided structures computed under a slowly varying envelope approximation for aneurysm A.

 Virtual stenting relies on a naive stent generator inspired by^72^, in which 2*n* wires are wrapped around the toroidal envelope parametrized by

3 $$\begin{aligned} ((r\cos \theta + R)\cos \frac{s}{R}\,,(r\cos \theta + R)\sin \frac{s}{R}\,,r\sin \theta )\,,\qquad (\theta , s)\in [0;2\pi ]\times [0;l]\,, \end{aligned}$$

where *r* and *R* are the minor and major radii of the torus, and *l* is its centerline length. The wire centerlines follow hhelical curves generated from a circular basis, that in turn provides the scaffold for the struts. Circular profiles are then extruded along the splines to generate the final wires with diameter $d=60\,\mu m$. Nominal heterogeneous functional parameters (yet homogeneous within a given group of wires) functional parameters, e.g., braiding angle, porosity (the percentage ratio of the wire-free surface area) and pore density (the number of pores per unit surface area) are obtained by braiding two by two parallel wires from $n_{j}$ initial positions uniformly distributed in each quadrant of the proximal end section (labelled counter-clockwise, adjusting the origin of azimuthal angle for the first quadrant of the cylinder to be mapped into the upper outer quadrant of the torus). In practice, all wire paths are actually computed under a slowly varying envelope approximation using

4 $$\begin{aligned} r(s)=r_{prox}\left( 1-\frac{s}{l}\right) + r_{dist}\frac{s}{l}, \end{aligned}$$

to fit the weak variations in the minor radius caused by the irregular patient-specific vascular geometry. This is because the variations for the cases documented herein (by about 11% relative to the average value) have been found to be well modelled by affine transformations, but more complex analytical functions can be specified as well. Examples of generated stents are given on Fig. 10.

### Computational domain and mesh

 The medical imaging data of both patients is segmented using the 3D Slicer software. The entire proximal portion of the parent artery visible in the images is reconstructed, for which 3D Slicer outputs point coordinates and connectivity of the centerline, together with the corresponding vessel radius (as defined by the minimal distance from the centerline to the vessel boundary) and curvature. A non-shrinking filter^73^ is used as an additional step of shape regularization to obtain smooth surface triangulation of the aneurysm lumen and connected vessel walls (which helps mitigate the effect of inner surface roughness). All vessels are truncated at some distance from the aneurysm bulge and extended with straight cylindrical pipes closed perpendicularly to their axis, to allow for flow development and ease the subsequent application of inflow/outflow boundary conditions.

For each patient, three-dimensional unstructured isotropic meshes of the vascular domain and stent devices are generated with the Gmsh software^74^, after which the vascular grid is finely and anisotropically refined with the method described in^75^. This allows evaluating any stent design sampled by DRL on the same vascular mesh, and thus yields considerable saving in the overall computational time. For the stent devices, 4 mesh points are allocated across any wire diameter, which is a reasonable compromise to assess feasibility while producing qualitative results to build on, as it yields a number of mesh elements that stands well below the few ten million elements reported in previous studies^54,55^. This in turn keeps the computational cost affordable, which is mandatory given that optimization requires evaluating the performance of hundreds of stent designs.

### Computational hemodynamics framework

 The blood flow is mathematically modelled after the three-dimensional incompressible Navier–Stokes equations

5 $$\begin{aligned} \nabla \cdot \varvec{u}=0\,,\qquad \qquad \rho (\partial _{t}\varvec{u}+ \varvec{u}\cdot \nabla \varvec{u})= \nabla \cdot (-p{{\textbf {I}}}+2\mu \varvec{\varepsilon }(\varvec{u}))\,, \end{aligned}$$

where $\varvec{u}$ is the velocity field, *p* is the pressure, $\varvec{\varepsilon }(\varvec{u})$ is the rate-of-deformation tensor, $\rho =$ 1050 kg/m$^3$ is the constant blood density, and $\mu$ is the non-Newtonian blood viscosity evaluated from the Carreau–Yasuda law using zero-shear rate viscosity $\mu _0=0.0456$ Pa.s, infinite-shear rate viscosity $\mu _\infty =0.00320$ Pa.s, relaxation time $\tau =10.03$ s, power law index $n=0.344$ and transition parameter $a=1.25$ (all values for a hematocrit of 40 % and a temperature of 37 $^\circ$C). The instantaneous wall shear stress whose peak value over a cardiac cycle is used for reward evaluation is computed as

6 $$\begin{aligned} \text {WSS}=\frac{3n+1}{4n}\mu \dot{\gamma }\delta _{\text {sac}}\,, \end{aligned}$$

where $\dot{\gamma }=(2\varvec{\varepsilon }(\varvec{u})\!:\!\varvec{\varepsilon }(\varvec{u}))^{1/2}$ is the wall shear rate defined as the second invariant of the rate-of-deformation tensor, $\delta _{\text {sac}}$ is a boolean representation of the aneurysm surface (obtained by embedding a portion of the adapted body-fitted grid truncated to remove the extra-aneurysmal domain), and the prefactor is the Weissenberg–Rabinowitsch correction for shear-thinning effects^76^. Since the elastic motion of the arterial wall is overlooked as a first approximation, simple open flow conditions are used, that consist of no-slip conditions at the solid nodes, zero-stress outflow conditions, and pulsatile, parabolic inflow condition

7 $$\begin{aligned} \varvec{u}=\frac{2Q(t)}{\pi r^2}\left( 1-\frac{||{\varvec{x}}||^2}{r^2}\right) \varvec{n}, \end{aligned}$$

where $||{\varvec{x}}||$ and $\varvec{n}$ are respectively the distance to the centerline and the normal vector in the inlet section of the parent artery, and *Q* is the time-dependent, volumetric flow rate adjusted at each time step to 2D-PCMRI measurements of the patients cross-sectionally averaged blood velocity (using linear regression from the two closest data points whenever the simulation and acquisition times do not coincide).

### Variational multiscale modeling

 A stabilized weak form of Eq. (5) is solved with a finite element variational multiscale method (VMS^77–79^). Such an approach consists in splitting the solution into coarse and fine-scale components, each corresponding to a different level of resolution. Only the large scales are fully represented and resolved at the discrete level. The fine scales are approximated in a way such that their effect into the large-scale equations is modelled after consistently derived source terms proportional to the residual of the resolved scale solution. Exhaustive details in^80^ regarding the derivation of the stabilized formulations lead to the following weak form for the large scale

8 $$\begin{aligned}&(\rho (\partial _t^{}\varvec{u}+\varvec{u}\cdot \nabla \varvec{u})\,,\,\varvec{w})+(2\mu \varvec{\varepsilon }(\varvec{u})\,,\,\varvec{\varepsilon }(\varvec{w})) -(p\,,\,\nabla \cdot \varvec{w})+(\nabla \cdot \varvec{u}\,,\,q)\nonumber \\&\quad =\sum _{K\in \mathcal {T}_h}\left[ (\tau _M\mathcal {R}_M\,,\,\varvec{u}\cdot \nabla \varvec{w}+\nabla q)_K +(\tau _C\mathcal {R}_C\,,\,\nabla \cdot \varvec{w})_K \right] , \end{aligned}$$

where $(\,,\,)$ is the $L^2$ inner product on the computational domain, $(\,,\,)_K$ is the inner product on element *K*, $\varvec{w}$ and *q* are relevant test functions for velocity and pressure, $\mathcal {R}_{C,M}$ are the governing equations residuals

9 $$\begin{aligned} -\mathcal {R}_C=\nabla \cdot \varvec{u}\,,\qquad \qquad -\mathcal {R}_M=\rho (\partial _t^{}\varvec{u}+\varvec{u}\cdot \nabla \varvec{u})+\nabla p\quad \end{aligned}$$

and $\tau _{C,M}$ are ad-hoc mesh-dependent stabilization parameters (comparable to local coefficients of proportionality) defined in^81,82^.

We solve Eq. (8) with an in-house VMS solver whose accuracy and reliability is assessed in a series of previous papers, see^82,83^ for a detailed mathematical formulation of the IVM in the context of finite element VMS methods, and^84,85^ for applications to non-Newtonian flows in complex geometry. Equal order, linear interpolation is used for spatial discretization of the velocity and pressure variables (as the inf-sup condition does not need to be satisfied due to the additional stabilization terms). Time-stepping is first-order accurate and combines explicit (for the VMS stabilization parameters), implicit (for the viscous, pressure and divergence terms), and semi-implicit integration schemes (for the time derivatives, convection terms and VMS source terms, using backward differentiation formula and Newton–Gregory backward polynomial). The time-step is set to 0.02 s, which allows distributing 40 and 46 points per cardiac cycle for aneurysm A and B, respectively. All linear systems are preconditioned with a block Jacobi method supplemented by an incomplete LU factorization, and solved with the GMRES algorithm, with tolerance threshold set to $10^{-6}$.

### Computational hemodynamics framework with deep reinforcement learning

**Figure 11:**
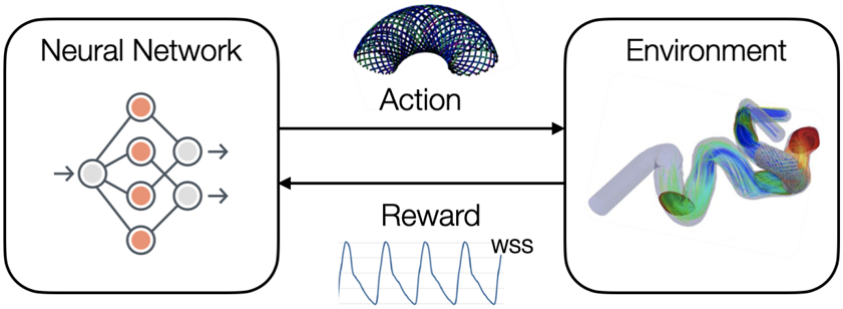
Sketch of the present DRL-CFD action-loop. The CFD hemodynamics environment and the DRL agent are coupled two-way through actions and rewards.

 The stent design is optimized solving a decision-making problem with reinforcement learning (RL), a process by which an agent learns to earn rewards through trial-and-error interaction with its environment. At each turn, the agent observes the state $s_t$ of the environment and takes an action $a_t$, that prompts both the transition to the next state $s_{t+1}$ and the reward received $r_t$. This repeats until the agent has learnt the succession of actions maximizing its cumulative reward over an episode (i.e., the reference unit for agent update, best understood as one instance of the scenario in which it takes actions). In the present context, the environment is a patient-specific CFD simulation of aneurysm hemodynamics after implantation of flow-diverting stent, that uses the computational hemodynamics framework described above. The agent is a policy represented by a deep neural network (a collection of artificial neurons that learns to represent a non-linear relation between input and output spaces, hence deep RL or DRL) trained with a RL algorithm, as reviewed in the next sections. The environment and the agent are coupled two-way, as illustrated in Fig. 11 : on the one hand, the actions sampled by the DRL agent (a set of five variables corresponding to four number of wires and a winding factor ) are used to generate the stent meshes immersed in the CFD simulation. On the other hand, the reward function needed by the agent to learn (here, the maximum value of MWSS) is obtained by post-processing of the CFD data.

### DRL agent

 A fully connected neural network is used, whose neurons are stacked in layers, each of which maps the biased weighted sum of their inputs through an activation function to produce their outputs and propagate the information forward from the input to the output layer via “hidden” layers (we use here 2 such hidden layers, each with 4 neurons feeding hyperbolic tangent activation functions). The network is trained with the single-step PPO algorithm, that learns a five-dimensional (four numbers of wires plus a winding factor) multivariate normal distribution whose mean and variance depend on the network weights and biases. Single-step PPO is a variation of the proximal policy optimization algorithm (PPO^25^) intended for situations where the optimal policy is independent of state, whose relevance for open-loop flow control is assessed in^48^. Just like PPO, it uses gradient ascent to maximize the surrogate loss

10 $$\begin{aligned} {\mathbb {E}}_{a\sim \pi _\theta } \left[ \min \left( \frac{\pi _\theta (a)}{{\pi _\theta }_{old} (a)} , 1+\epsilon {{\,\mathrm{sgn}\,}}{\left( \widehat{A}^{\pi _\theta }(a)\right) }\right) \widehat{A}^{\pi _\theta } (a)\right] \,, \end{aligned}$$

where $\pi _\theta (a)$ is the policy, i.e., a probability distribution of actions $\pi _\theta (a)$ parameterized by a set of free parameters $\theta$ (here the weight and biases of the deep neural network) that determines the agent behaviour, $\widehat{A}^{\pi _\theta }$ is a biased estimator of the advantage function $A^{\pi _\theta }$ measuring the gain of taking action *a* over the average value (here its normalization to zero mean and unit variance), and $\epsilon$ is a clipping range defining how far away the new policy is allowed to go from the old. A positive (resp. negative) advantage increases (resp. decreases) the probability of taking action *a*, but always by a proportion smaller than $\epsilon$, otherwise the min kicks in (10) and its argument hits a ceiling of $1+\epsilon$ (resp. a floor of $1-\epsilon$). This conservatism inherited from the parent algorithm ensures that the current and new policies behave similarly (which prevents the agent from falling off a cliff and restarting with a locally bad policy, in which case the performance may collapse drastically and never recover). Another trait shared by the two algorithms is the lack of necessity for assumptions regarding the optimization problem to be solved and for fine-tuning of the network hyper-parameters (i.e., those parameters not learnt from data).

Where the two methods differ is that PPO seeks the optimal set of actions $a^\star$ earning the largest possible reward, while single-step PPO seeks the optimal state-action mapping $f_{\theta ^\star }$ such that $a^\star = f_{\theta ^\star } (s_0)$, where $s_0$ denotes some input state consistently fed to the agent for the optimal policy to eventually embody the transformation from $s_0$ to $a^\star$. Starting from a random mapping $f_{\theta _0}$ from $s_0$ to the policy determined by the free parameters initialization, the agent gets one attempt per episode at finding the optimal (i.e., it interacts with the environment only once per episode) before updating the policy. Another subtle difference is that PPO is actor-critic, i.e., it features an actor network that learns the policy, and a critic network that learns to estimate the advantage. Single-step PPO works without knowledge of the critic evaluations (and is thus not actor-critic) because the trajectory of state and actions consists of a single pair. The discount factor adjusting the trade-off between immediate and future rewards can thus be set to $\gamma =1$, in which case the advantage reduces to the whitened reward^48^.

**Figure 12:**
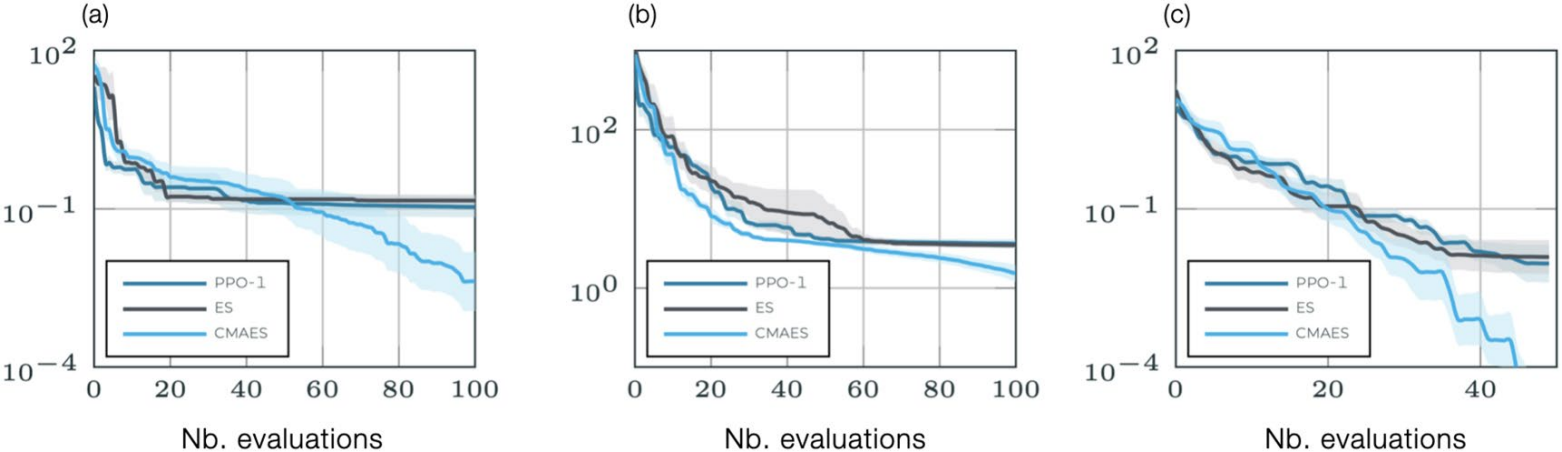
Benchmark minimization problems for the (**a**) two- and (**b**) five-dimensional Rosenbrock functions, and (**c**) the two-dimensional Branin function, using the present PPO-1 algorithm and reference ($\mu$-$\lambda$)-ES and CMA-ES evolutionary algorithms.

Our single step PPO method is based on the default open-source implementation of Stable Baselines (https://github.com/openai/baselines/tree/master/baselines/ppo2), for which a custom OpenAI environment has been designed with the Gym library^86^. We have updated and connected the original code with our CFD library for simple reading and writing of the results (the code is shared publicly using the following link : https://github.com/jviquerat/pbo). The convergence properties are illustrated in Fig. 12 for a minimization test problem of two- and five-dimensional Rosenbrock functions, whose global minimum is notoriously difficult to catch for optimization algorithms and two-dimensional Branin function, that has two identical global minima. For this case, the single-step PPO-1 algorithm is benchmarked against classical ($\mu$-$\lambda$)-ES and CMA-ES evolutionary methods, all implemented in in-house production codes. To ensure a fair comparison, the initial parameters and starting points are identical for all methods. All runs are afforded the same budget, namely 500 evaluations (20 episodes with 5 parallel environments in PPO-1, 20 generations with 5 individuals per generation in evolutionary algorithms) for Rosenbrock and 50 evaluations for Branin (10 episodes/generations with 5 parallel environments/individuals per generation). A large initial standard deviation is used by default, to ensure a good exploration of the optimization domain. Finally, in order to emphasize flexibility and generalizability, all PPO runs are tackled without fine-tuning of the algorithm, i.e., all runs use the same meta-parameters as in Table 1. Performances are averaged over 10 runs, with standard deviations shown as the light shade around. As could have been expected, the search efficiency of CMA-ES yields the best overall performance, which reflects the benefit of efficiently elongating the research area with respect to the local shape of the cost function. Among isotropic exploration methods, PPO-1 achieves final cost levels similar to ($\mu$-$\lambda$)-ES, with faster convergence and better performance at intermediate stages (the final performance level ultimately saturates for the Rosenbrock function because the minimum is in a long, narrow valley, and PPO-1/($\mu$-$\lambda$)-ES use isotropically sampled approximations of the descent direction). The general picture to be drawn is that (1) PPO-1 exhibits strong performance compared to methods relying on similar isotropic search distributions, and (2) anisotropic search distributions are mandatory to outperform more advanced methods on a consistent basis, an issue that is being addressed in current research efforts by the authors^87^.

### Parallel data collection

 In practice, actions are distributed to several environments running in parallel, each of which executes a self-contained MPI-parallel CFD simulation and feeds data to the DRL algorithm (hence, two levels of parallelism related to the environment and the computing architecture). All simulations are performed on a workstation of AMD EPYC 7502 processors. The algorithm waits for the simulations running in all parallel environments to complete, then shuffles and splits the rewards data set collected from all environments into several buffers (or mini-batches) used sequentially to compute the loss and perform a network update. This repeats for several epochs, i.e., several full passes of the training algorithm over the entire data set (which ultimately makes the algorithm slightly off-policy, since the policy network ends up being trained on samples generated by older policies). This simple parallelization technique is key to using DRL in the context of flow control applications, as estimating accurately the policy gradient requires assessing a sufficient number of actions drawn from the current policy, hence a large computational burden associated to reward computations for high-dimensional fluid dynamics problems (typically, the cost of a single call to the CFD solver times the number of evaluations required). In the same vein, it should be noted that the common practice in DRL studies to gain insight into the performances of the selected algorithm by averaging results over multiple independent training runs with different random seeds is not tractable, as it would trigger a prohibitively large CPU cost. The same random seeds have thus been deliberately used over the whole course of the study to ensure a minimal level of performance comparison between the two cases.

**Table 1:** PPO hyper parameters.

32	Nb. epochs
8	Nb. environments
2	Size of mini-batches
$5\times 10^{-3}$	Learning rate
0.3	Clipping range

## Data Availability

The datasets generated and analysed during the current study are not publicly available due the fact that they constitute an excerpt of research in progress but are available from the corresponding author on reasonable request.
